# Cohort study examining tamoxifen adherence and its relationship to mortality in women with breast cancer

**DOI:** 10.1038/sj.bjc.6604758

**Published:** 2008-11-04

**Authors:** C McCowan, J Shearer, P T Donnan, J A Dewar, M Crilly, A M Thompson, T P Fahey

**Affiliations:** 1 Division of Community Health Sciences, University of Dundee, MacKenzie Building, Kirsty Semple Way, Dundee DD2 4BF, UK; 2 Department of Radiotherapy and Oncology, Ninewells Hospital and Medical School, Dundee DD1 9SY, UK; 3 Department of Public Health, University of Aberdeen, School of Medicine, Polwarth Building, Aberdeen AB25 2ZD, UK; 4 Department of Surgery and Molecular Oncology, University of Dundee, Ninewells Hospital and Medical School, Dundee DD1 9SY, UK; 5 Department of General Practice, Royal College of Surgeons in Ireland, 120 St Stephens Green, Dublin 2, Ireland

**Keywords:** breast cancer, tamoxifen, adherence, mortality, community

## Abstract

Increasing duration of tamoxifen therapy improves survival in women with breast cancer but the impact of adherence to tamoxifen on mortality is unclear. This study investigated whether women prescribed tamoxifen after surgery for breast cancer adhered to their prescription and whether adherence influenced survival. A retrospective cohort study of all women with incident breast cancer in the Tayside region of Scotland between 1993 and 2002 was linked to encashed prescription records to calculate adherence to tamoxifen. Survival analysis was used to determine the effect of adherence on all-cause mortality. In all 2080 patients formed the study cohort with 1633 (79%) prescribed tamoxifen. The median duration of use was 2.42 years (IQR=1.04–4.89 years). Longer duration was associated with better survival but this varied over time. The hazard ratio for mortality in relation to duration at 2.4 years was 0.85, 95% CI=0.83–0.87. Median adherence to tamoxifen was 93% (interquartile range=84–100%). Adherence <80% was associated with poorer survival, hazard ratio 1.10, 95% CI=1.001–1.21. Persistence with tamoxifen was modest with only 49% continuing therapy for 5 years of those followed up for 5 years or more. Increased duration of tamoxifen reduces the risk of death, although one in two women do not complete the recommended 5-year course of treatment. A significant proportion of women have low adherence to tamoxifen and are at increased risk of death.

Tamoxifen is an important adjuvant therapy for patients with breast cancer (Osborne, 1998) and reduces the breast cancer mortality rate over 15 years by 31% in women with oestrogen receptor-positive early breast cancers. (Early Breast Cancer Trialists’ Collaborative, 2005) Tamoxifen (20 mg once daily) is recommended for patients for 5 years adjuvant therapy (Early Breast Cancer Trialists’ Collaborative Group, 2004) to provide maximum benefit (Fisher *et al*, 1996; Stewart *et al*, 1996; Burstein and Winer, 2000).

Adherence to a prescribed medication can be defined as: ‘the extent to which a patient's behaviour … coincides with medical or health advice’ (McDonald *et al*, 2002). Non-adherence to prescribed medication for patients with chronic conditions is a major public health issue. The level of non-adherence reported by studies is large, up to 50%, and many studies have shown worse clinical outcomes associated with low adherence (DiMatteo, 2004).

The literature for tamoxifen deals with three different aspects of adherence to medication. The first, duration of tamoxifen therapy has an effect on disease recurrence and mortality from breast cancer with 5 years being the recommended treatment period (Swedish Breast Cancer Cooperative Group, 1996; Fisher *et al*, 2001; Yood *et al*, 2008). A second closely related aspect of adherence is persistence or continuation of therapy where the period that a patient continues to take the medication before a pre-specified break in medication is measured. This differs from duration in that if a patient restarts therapy after a break of the specified length this additional use of medication is ignored. Several recent studies have reported non-persistence with tamoxifen therapy. The first reported cumulative non-persistence for tamoxifen in a cohort of 2816 women at 22.1% after 1 year and 35.2% at 3.5 years (Barron *et al*, 2007). The second followed 961 women over 5 years and found that 49% stopped taking their tamoxifen before the recommended 5-year treatment period (Owusu *et al*, 2008). An earlier study reported 17% of women aged 65+ years had stopped treatment within 2 years (Fink *et al*, 2004). However, none of these studies assessed the impact of failure to persist with therapy on mortality or the independent effect of non-adherence in patients taking tamoxifen.

The last aspect of adherence to tamoxifen is whether the patients take the medication consistently on a daily basis as prescribed. A recent review concluded that this aspect of non-adherence to adjuvant medication may limit the efficacy of breast cancer treatment, although there was limited evidence for this (Chlebowski *et al*, 2006). Adherence to adjuvant tamoxifen during clinical trials is high, with reported levels of 94–100% (Fisher *et al*, 1994, 1996; Dewar *et al*, 1996). Community-based studies found that 23% of patients failed to take tamoxifen in 1 out of 5 days with adherence falling from 89 to 50% over 4 years (Partridge *et al*, 2003) and that deviations from prescribed tamoxifen therapy were common (Demissie *et al*, 2001). A small survey on self reported adherence in 53 women found that 62% acknowledged having missed at least one dose of tamoxifen in the previous 6 months, with 24% missing at least one dose per week (Murthy *et al*, 2002). These studies suggest that non-adherence to tamoxifen is underestimated and further understanding of the relationship to mortality is required. No previous study has examined the overall effect on all-cause mortality of duration of use, adherence to and persistence with tamoxifen either in the hospital or community setting.

The aim of this study was to investigate adherence to tamoxifen for all women with breast cancer drawn from a defined geographical population in Tayside, Scotland. The objectives were to:

Describe the duration of use, adherence to and persistence with tamoxifen in a geographically defined population of women.

Investigate whether duration and adherence to tamoxifen influenced all-cause mortality after adjusting for important clinical and demographic factors.

## Materials and methods

### Identification of study cohort

Women resident in Tayside registered with the Cancer Registry as having breast cancer or with an initial hospital admission for breast cancer from 1993 to January 2002 were identified. Only those women who were resident for the entire period of the study or until death were included. Patients who had prescribing or hospital admission records, an audit record and a Cancer Registry record within the study period comprised the population of interest. Every patient registered with a general practitioner in Tayside is assigned a 10 digit unique patient identifier, the Community Health Index number (CHI number) used in all NHS encounters. The CHI number, which includes the patient's date of birth, allows linkage of health-related datasets providing a unique resource combining information on dispensed prescribing with detailed clinical data at the individual patient level.

### Study covariates

Date of diagnosis was recorded from either the Cancer Registry or the clinical audit records and age at diagnosis was then calculated. Duration of breast cancer was calculated from the date of diagnosis to the date of death or until 7 January 2002, which was the study cutoff point. Each patient had a Carstairs score (a measure of deprivation (Carstairs and Morris, 1990)) and subsequent category calculated based on their home postcode using census data from 2001. There were only two patients in Carstairs category 7, the most deprived grouping, and so categories 6 and 7 were grouped together for analysis.

A Charlson's co-morbidity index, which included a category for presence of cancer, was derived for each patient using standard procedures and the ICD codes version 9 (Deyo *et al*, 1992) and 10 (Sundararajan *et al*, 2004) in the hospital admission records. The encashed prescribing records were also examined and the Charlson disease groups were flagged for patients who were receiving medication for respiratory disease, AIDS, peptic ulcers, cancer, metastatic tumours, diabetes, myocardial infarction, connective tissue disorders and dementia. Charlson Index scores were calculated and categorised into three groups with low (0–2), medium (3–5) and high morbidity (6+). The number of different preparations prescribed to patients’ for the 6 months prior to diagnosis was counted and categorised into four groups, 0, 1–2, 3–5 or 6+. The characteristics of the tumour on presentation were recorded using clinical TNM categories (tumour size, presence of regional node metastasis and distant metastases), pathological grading, pathological description of axillary node metastasis and oestrogen receptor status. Where conflicts existed between the cancer registry and clinical audit records, the audit records were used as the primary source of information.

### Tamoxifen adherence

**Figure 1:**
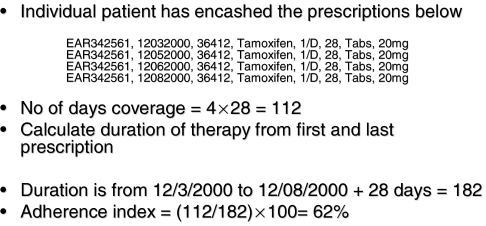
Calculation of adherence and duration.

Each prescription for tamoxifen was analysed and the number of days covered by that prescription recorded from the number of dispensed tablets and daily dose. The duration of tamoxifen therapy was calculated from the number of days between the first and last prescription and the coverage of the last prescription. The adherence index for each patient was calculated by: summing the coverage for all the prescriptions for each patient, dividing it by the duration, and then converting this to a percentage (see Figure 1). The adherence index was calculated across the entire duration of therapy whether this exceeded 5 years or not. Based on the existing literature (Murthy *et al*, 2002; Wei *et al*, 2002; Partridge *et al*, 2003) patients with an adherence index less than 80% were deemed to have ‘low adherence’ (Osterberg and Blaschke, 2005).

To allow a direct comparison with previous studies, (Barron *et al*, 2007) persistence was calculated as the length of time from first prescription to a break of at least 180 days before the completion of 5 years of therapy. Patients who continued treatment had their length of persistence calculated using either the date of death or the end of the study period.

### Outcome variable

The main outcome measure was all-cause mortality recorded on the General Registry Office death certificate.

### Statistical analysis

Data were described as number of subjects (percentages) for categorical variables and mean (s.d.) for continuous variables. Where continuous variables did not follow a normal distribution, tested using the Shapiro–Wilks test for skewness, the median and interquartile range were reported. *χ*^2^ tests for trend (*χ*^2^ trend, d.f., *P*) were reported for differences in distribution of the population with *n* categories. Cox Proportional Hazards models were utilised to estimate hazard ratios and 95% confidence intervals for each unadjusted and adjusted covariate for all-cause mortality. Patients were followed up from date of diagnosis or commencement of tamoxifen until time of death or the end of the study.

Multivariable results are presented as hazard ratios and 95% confidence intervals. Covariates were included in the multivariate model if they were deemed to be of clinical significance or had univariable *P*-value <0.2. The proportional hazards assumption was assessed using trend-tests of the Schoenfeld residuals and those which failed the assumption or which were deemed to be time-dependent were entered as continuous time-dependent covariates (Bradburn *et al*, 2003).

In a community-based, non-randomised study many known and unknown factors may determine who receives tamoxifen, which can potentially bias results. A propensity score estimating the probability of a patient receiving tamoxifen was calculated using a logistic regression model for age, social class, pathological tumour characteristics, Charlson's index and co-prescribing (Wang and Donnan, 2001). This was added to the model to adjust for propensity to receive tamoxifen and so reduce bias in the survival analysis. All statistical analyses were performed using Stata version 9.

The study was granted approval from the Tayside Committee on Medical Research Ethics and the Caldicott Guardian.

## Results

A total of 2184 patients were identified with prescribing, hospital admission records for breast cancer surgery, cancer audit and cancer registry records for the period of the study. Patients who were known to have had cancer before 1993, or who received tamoxifen more than 6 months prior to their diagnosis or those diagnosed with ductal carcinoma *in situ* alone were excluded. This left a total of 2080 patients whose records were analysed within this study. The mean age at diagnosis was 61.4 (s.d.=14.02) and patients were followed up for a total of 7619 person years with a median follow-up of 3.16 years (interquartile range=1.38–5.72 years). A total of 511 deaths (25%) were recorded.

### Descriptive statistics for duration, adherence and persistence with tamoxifen

Tamoxifen was prescribed as therapy to 1633 (79%) patients of whom 414 (25%) died during the study. The median duration of tamoxifen in the study was 2.42 years (interquartile range=1.04–4.89 years). Patients were generally highly adherent to their medication during the course of treatment with a median adherence of 93% (interquartile range=84–100%). There were 315 (19%) patients with low adherence of less than 80%. There was no difference in low adherence by social class (*P*=0.96) but there was a trend for a higher proportion of younger women to have low adherence (*P*<0.001).

However, 411 (33%) patients prescribed tamoxifen discontinued their medication before completing 5 years of treatment. Within 1 year of commencing tamoxifen 10% of patients had discontinued treatment, for patients followed for 2 years or more 19% patients had discontinued treatment, 32% patients for those followed for three and a half years or more and 51% patients of those followed for 5 years plus.

### Comparison of tamoxifen users and non users

The distribution of age, social class, Charlson's index and co-prescribing is presented in Table 1. Women in the younger age groups were less likely to receive tamoxifen than older patients (*P*<0.001); social class categories 2 and 3 had the highest proportion of women not receiving the drug (*P*=0.011) and patients who received tamoxifen had higher levels of co-prescribing (*P*<0.001) and greater Charlson's Index scores (*P*<0.001).

### Patient characteristics and tamoxifen usage

The clinical and pathological characteristics of the cancer at diagnosis are shown in Table 2. There were no differences in clinical tumour stage (*P*=0.86) between tamoxifen users and those who did not receive the drug. However, patients were less likely to receive tamoxifen therapy if they had positive clinical nodes at initial diagnosis (*P*=0.001), if metastases were present (*P*=0.03); if tumour grade was worse (*P*<0.001), if patients had positive nodes on pathology (*P*<0.001) or if they had negative oestrogen receptor status (*P*<0.001).

### Cox proportional hazards model for all-cause mortality

An initial multivariate model was created to investigate the effect of tamoxifen use and showed that patients not prescribed tamoxifen were at significantly higher risk of death than patients who were prescribed tamoxifen after allowing for all other covariates (HR=1.36, 95% CI=1.05–1.76).

A subsequent multivariate model investigated the adjusted effect of covariates for those patients who used tamoxifen. Adjusting for all factors, increasing age, increasing tumour grade, positive or unknown pathological node status and negative or unknown oestrogen receptor status increased the risk of death. A Charlson's Index score of 6 or more also increased the risk of death over time, at the median duration of 2.4 years, the hazard ratio increased by 1.59, 95% CI=1.14–2.21. Duration of tamoxifen use was also associated with better survival after allowing for other covariates and the hazard ratio at a given time could be calculated using the equation HR=exp(−0.065 × follow-up time). At 2.4 years, the median duration, the hazard ratio was 0.85, 95% CI=0.83–0.87 (see Table 3).

Patients with an adherence index of <80% had an increased hazard of death calculated using the equation HR=exp(0.04 × follow-up time). At the median duration of tamoxifen use of 2.4 years the hazard ratio for low adherence was 1.10, 95% CI=1.001–1.21 (Table 3).

Tamoxifen is not recommended for use in patients with oestrogen receptor negative tumours, although within our study population it was used with this patient group. Restricting the analysis to patients with ER +ve or ER status unknown was associated with a significantly increased hazard of death for adherence below 80%, HR=exp(0.05 × follow-up time), 95% CI 0.01–0.10, the hazard ratio at the median duration being 1.13, 95% CI=1.01–1.26.

## Discussion

This study suggests that adherence to taking daily tamoxifen in the community setting is high, over four-fifths of women prescribed tamoxifen had an adherence of 80% or above during the course of treatment. However, cumulative persistence with tamoxifen treatment is low, with 51% of the patient population followed for 5 years or more discontinuing treatment by 5 years. The study defined high adherence at 80% or over and there was a significant increase in hazard of death for patients with adherence beneath that level. This suggests that the longer a patient has low adherence the greater their increase in hazard of death. Patients who were prescribed tamoxifen had better survival after adjusting for other factors than those who did not, and the duration of tamoxifen use was also seen to reduce the hazard of death.

This study supports the use of tamoxifen in the community setting as an adjuvant therapy that reduces mortality in women with breast cancer. Though the reduction in hazard in this community-based study is lower, the higher survival figures reported in randomised controlled trials may reflect different inclusion criteria or some selection bias in terms of recruiting ‘healthier’ patients with less co-morbid illness to these trials.

In terms of generalisability, the study population comprised of women who had an initial attendance at a hospital for breast cancer, had an accompanying cancer registry record entry and a history of prescribing. The women also needed to be resident in the region served by the breast cancer service for the entire study period or until death to ensure that there was no loss to follow-up. Thus the study cohort of 2184 women accounts for 85% of potential patients.

Around three-quarters of breast cancer patients in Tayside received tamoxifen as adjuvant therapy, as reported elsewhere (Demissie *et al*, 2001). Approximately one-fifth of tamoxifen users had adherence below 80% similar to studies elsewhere (Murthy *et al*, 2002; Partridge *et al*, 2003). Non-persistence with tamoxifen therapy at 1 year differed from the figure reported in the study by Barron *et al* (2007) but at three and a half years was at a similar level and was similar to the figure reported by Fink *et al* (2004) at 2 years and Owusu *et al* (2008) at 5 years. Hence, the characteristics of tamoxifen use, adherence and persistence in this population is similar to those from other published work. This study concentrated on the duration of tamoxifen use and adherence to taking it on a prescribed daily basis, as this is a more complete marker of use than non-persistence. What is added to the existing literature is the linkage between low adherence and increased all-cause mortality.

The use of the propensity score within the final model helps reduce potential selection bias (Wang and Donnan, 2001) because of the use of observational data, where clinicians may choose patients with a lower risk of mortality to receive tamoxifen. However, our analysis holds after allowing for the predisposition of a patient to receive tamoxifen. With 79% of patients receiving tamoxifen it is unlikely that they were selected as having better survival prospects.

In terms of potential shortcomings, this study measured the adherence to medication by looking at the encashment of prescriptions. Although there are advantages and disadvantages to various techniques for measuring adherence, (Osterberg and Blaschke, 2005) and whereas we cannot tell if patients actually took the medication, breast cancer patients are probably motivated to be adherent, as reflected in the high levels of adherence reported by this and other studies. Tayside has a closed pharmacy system as all encashed prescriptions are returned to a central government office for reimbursement and this allowed us to measure adherence objectively across the whole population.

There was approximately 1 in 5 patients with less than 80% adherence, which means this study had low power to determine if adherence has an effect on mortality. In addition, it is now appreciated that ER-negative patients (200 in this study) do not benefit from adjuvant tamoxifen and would also reduce power. The study aimed to look at the effect of adherence to tamoxifen on survival in a broad range of women with breast cancer-prescribed tamoxifen in the community. Consequently, we included patients in the study cohort who would not have been eligible for inclusion in trials or where current treatment guidelines would not recommend its use. There were 105 patients with clinical metastases who received tamoxifen and such women may have had a different motivation to take their medication than those receiving it as adjuvant therapy. As the aim of our study was to assess the effect of adherence to tamoxifen in the community, using a comprehensive prescribing dataset to monitor long-term adherence, we intentionally included both patients with clinical metastases and also patients who were ER negative in our analysis. The effect of adherence to tamoxifen on survival was still evident in subsequent analysis performed on the cohort with these patient groups excluded.

This study could not explain why some women were not adherent to or discontinued their medication, although it suggested younger women were more likely to have low adherence. Other work agrees with this finding and also suggests side effects or a perception of low benefits from taking medication leads to lower adherence in younger women (Fink *et al*, 2004; Grunfeld *et al*, 2005; Chlebowski *et al*, 2006).

The study did not report on chemotherapy or radiotherapy use. Treatment in Tayside used standard practises for both treatments over the study period so it seems unlikely that there could be any systematic difference in chemotherapy or radiotherapy use between any of the groups of interest. Furthermore, radiotherapy affects mortality only after 10–15 years so it would not have been a factor within our analysis (Clarke *et al*, 2005).

Recurrence of breast cancer was also omitted because of the difficulties in ascertaining recurrence within a community setting. The routine datasets used do not record cancer recurrences directly and so a proxy measure would have been required which we decided against. Although this would not affect the main outcome of mortality we may have overestimated the number of women who discontinue their medication.

Lastly, the outcome used within the study was all-cause mortality selected as the accurate recording of cause of death has been questioned in the literature (Clark *et al*, 2003).

This study confirms that around 20% of patients have an adherence level, <80%, the equivalent of missing at least one tablet every 5 days. Our findings suggest that adherence to tamoxifen beneath this level has a negative effect on survival. The relatively long half-life of tamoxifen (Osborne, 1998) may mean that missing an occasional tablet is less of an issue and that the patients will still benefit from tamoxifen therapy. Aromatase inhibitors are increasingly used as adjuvant therapy but have a shorter half-life than tamoxifen and so strict adherence to this medication regimen should be emphasised as the occasional missed dose may have a greater detrimental effect on survival.

## Conclusion

Patients who are prescribed adjuvant tamoxifen in the community have a lower risk of death with increased duration of use further reducing the risk. Cumulative non-persistence with tamoxifen therapy occurs in nearly half of patients prescribed tamoxifen. Conversely, in those who continue to take tamoxifen adherence is generally high, but there is a significant proportion of women with low adherence who are at greater risk of death. Patients need to be encouraged to continue their medication for the full 5-year recommended period to ensure their best chance of survival.

## Figures and Tables

**Table 1:** Descriptive statistics of cohort- age, social class, Charlson's index and co-prescribing

	**Patients with tamoxifen (%)**	**No tamoxifen (%)**	**Wald *χ*^2^ test (Wald, d.f., *P*)**
Numbers	1633 (79)	447 (21)	
			
*Age group*			
<40	84 (5)	37 (8)	22.6, 5, *P*<0.001
40–49	237 (15)	94 (21)	
50–59	435 (27)	112 (25)	
60–69	365 (22)	95 (21)	
70–79	331 (20)	77 (17)	
80+	181 (11)	32 (7)	
			
*Carstairs category*			
1	152 (9)	29 (7)	14.9, 5, *P*=0.011
2	327 (20)	108 (24)	
3	443 (27)	145 (32)	
4	306 (19)	66 (15)	
5	164 (10)	34 (8)	
6/7	237 (15)	64 (14)	
			
*Number of prescriptions at diagnosis*			
0	500 (31)	254 (57)	104.9, 3, *P*<0.001
1–2	457 (28)	77 (17)	
3–5	397 (24)	63 (14)	
6+	279 (17)	53 (12)	
			
*Charlson's index*			
0–2	476 (29)	198 (44)	37.9, 2, *P*<0.001
3–5	510 (31)	100 (22)	
6+	647 (40)	149 (33)	

**Table 2:** Clinical and pathological characteristics of cancer at initial diagnosis

	**Patients with tamoxifen (%)**	**No tamoxifen (%)**	**Wald *χ*^2^ test (Wald, d.f., *P*)**
Numbers	1633 (79)	447 (21)	
			
*Tumour stage*			
1	440 (27)	119 (27)	1.3, 4, *P*=0.86
2	522 (32)	136 (30)	
3	118 (7)	36 (8)	
4	141 (9)	35 (8)	
Unknown	412 (25)	121 (27)	
			
*Clinical nodes*			
0	1144 (70)	316 (71)	17.7, 3, *P*=0.001
1	230 (14)	74 (17)	
2	34 (2)	20 (4)	
Unknown	225 (14)	37 (8)	
			
*Clinical metastases*			
No	1354 (83)	365 (82)	7.1, 2, *P*=0.03
Yes	72 (4)	33 (7)	
Unknown	207 (13)	49 (11)	
			
*Tumour grade*			
1	221 (14)	48 (11)	86.7, 3, *P*<0.001
2	545 (33)	128 (29)	
3	363 (22)	194 (43)	
Unknown	504 (31)	77 (17)	
			
*Oestrogen receptor status*			
Positive	869 (53)	142 (32)	227.1, 2, *P*<0.001
Negative	199 (12)	195 (43)	
Unknown	565 (35)	110 (25)	
			
*Pathological nodes*			
Negative	754 (46)	230 (52)	16.4, 2, *P*<0.001
Positive	446 (27)	140 (31)	
Unknown	433 (27)	77 (17)	

**Table 3:** Multivariate association between covariates and all cause mortality

	**Adjusted for other covariates**		
**Predictor**	**HR**	**95% CI**	
Age (10 years)	1.15	1.05–1.27	*P*=0.004
			
*Carstairs category*			
1 (most affluent)	1.0		
2	0.86	0.56–1.32	*P*=0.486
3	1.03	0.69–1.54	*P*=0.878
4	1.23	0.81–1.86	*P*=0.333
5	1.04	0.66–1.65	*P*=0.851
6/7 (most deprived)	1.27	0.82–1.97	*P*=0.353
			
*Charlson's index (at 2.4 years)*			
0–2	1.0		
3–5	1.21	0.83–1.78	*P*=0.325
6+	1.59	1.14–2.21	*P*=0.006
			
*Number of prescriptions at diagnosis*			
0	1.0		
1–2	0.75	0.46–1.23	*P*=0.253
3–5	0.56	0.22–1.22	*P*=0.143
6+	0.44	0.16–1.18	*P*=0.104
			
*Tumour grade*			
1			
2	2.49	1.24–4.98	*P*=0.010
3	4.03	2.01–8.08	*P*<0.001
Unknown	2.22	0.89–5.54	*P*=0.089
			
*Oestrogen receptor status*			
Positive	1.0		
Negative	1.96	1.46–2.62	*P*<0.001
Unknown	1.47	1.10–1.97	*P*=0.009
			
*Pathological nodes*			
Negative	1.0		
Positive	2.05	1.54–2.72	*P*<0.001
Unknown	2.19	1.57–3.04	*P*<0.001
Propensity score	0.01	0.00–3.30	*P*=0.106
Duration of tamoxifen (at 2.4 years)	0.85	0.83–0.87	*P*<0.001
Adherence <80% (at 2.4 years)	1.10	1.001–1.21	*P*=0.046
